# Altering bioelectricity on inhibition of human breast cancer cells

**DOI:** 10.1186/s12935-016-0348-8

**Published:** 2016-09-22

**Authors:** Seher Berzingi, Mackenzie Newman, Han-Gang Yu

**Affiliations:** 1 Department of Biology, West Virginia University, Morgantown, WV 26506 USA; 2 Department of Physiology & Pharmacology, West Virginia University, Morgantown, WV 26506 USA

**Keywords:** Membrane potential, Voltage-gated calcium channels, Voltage-gated potassium channels, Verapamil, TEA, Caspase-3, Caspase-9

## Abstract

**Background:**

Membrane depolarization is associated with breast cancer. Depolarization-activated voltage-gated ion channels are directly implicated in the initiation, proliferation, and metastasis of breast cancer.

**Methods:**

In this study, the role of voltage-gated potassium and calcium ion channel modulation was explored in two different invasive ductal human carcinoma cell lines, MDA-MB-231 (triple-negative) and MCF7 (estrogen-receptor-positive).

**Results:**

Resting membrane potential is more depolarized in MCF7 and MDA-MB-231 cells compared to normal human mammary epithelial cells. Increasing extracellular potassium concentration up to 50 mM depolarized membrane potential and greatly increased cell growth. Tetraethylammonium (TEA), a non-specific blocker of voltage-gated potassium channels, stimulated growth of MCF7 cells (control group grew by 201 %, 1 mM TEA group grew 376 %). Depolarization-induced calcium influx was hypothesized as a requirement for growth of human breast cancer. Removing calcium from culture medium stopped growth of MDA and MCF7 cells, leading to cell death after 1 week. Verapamil, a blocker of voltage-gated calcium channels clinically used in treating hypertension and coronary disease, inhibited growth of MDA cells at low concentration (10–20 μM) by 73 and 92 % after 1 and 2 days, respectively. At high concentration (100 μM), verapamil killed >90 % of MDA and MCF7 cells after 1 day. Immunoblotting experiments demonstrated that an increased expression of caspase-3, critical in apoptosis signaling, positively correlated with verapamil concentration in MDA cells. In MCF7, caspase-9 expression is increased in response to verapamil.

**Conclusions:**

Our results support our hypotheses that membrane depolarization and depolarization-induced calcium influx stimulate proliferation of human breast cancer cells, independently of cancer subtypes. The underlying mechanism of verapamil-induced cell death involves different caspases in MCF7 and MDA-MB-231. These data suggest that voltage-gated potassium and calcium channels may be putative targets for pharmaceutical remediation in human invasive ductal carcinomas.

## Background

Breast cancer is a leading cancer in women. In 2015, 231,840 new cases of female invasive cases were diagnosed in the US (American Cancer Society, Surveillance Research, 2015). Traditionally, there are three clinical subtypes of breast cancer: estrogen or progesterone receptor-positive (ER+ or PR+), human epidermal growth factor receptor 2 positive (HER2+), and triple negative (ER−, PR−, HER2−). Recently, based on genetic profiling, revised clinical classifications suggested five subtypes: luminal A, luminal B, basal, claudin-low, and HER2 [1].

Invasive ductal carcinoma (IDC) is the most prevalent type of breast cancer. For this study, we chose to use MCF7 (ER+, PR+, or luminal A) and MDA-MB-231 (triple-negative or claudin-low) cells due to their prevalence in contemporary cancer research, well-defined characteristics, and similarity to in vivo IDC development when cultured. These two cell lines, along with T-47D, account for over two-thirds of all abstracts on breast cancer growth studies [2]. The MCF7 cell line has become the most widely-used breast cancer line since its establishment in 1973. MDA-MB-231 cells exhibit a more aggressive phenotype in contrast to MCF7 cells [3] and are therefore a model of intractable IDC.

Bioelectrical potentials depend on ion flux in biological cells. Due to an asymmetrical distribution of ions across the plasma membrane, ions constantly flow in and out of cell membranes through their transporters: ion channels, pumps and exchangers [4]. Gating of ion channels results in changes in resting membrane potential which is essential for both normal cell and cancer development [4]. The roles of voltage-gated calcium channels, sodium channels, and potassium channels in cancer cell proliferation, invasion, and metastasis have only recently been demonstrated [4–9].

Breast cancer cells isolated from patients have a resting membrane potential of −13 mV [10]. In normal human mammary epithelial cells (HMEC), the resting membrane potential is −60 mV [10]. Membrane depolarization has been hypothesized to stimulate cell proliferation [4]. Membrane depolarization activates voltage-gated calcium channels and causes an influx of Ca^2+^ through T-type voltage-gated calcium channels in human breast cancer cells [11]. Calcium channels play a pivotal role in proliferation and tumorigenesis of the cell, yet the underlying mechanism is not completely understood [12]. T-type voltage-gated calcium channels have been reported to be overexpressed in MCF7 and play an important role in cell proliferation [13]. Potassium channel blockers such as 4-aminopyridine mimic the effects of calcium channel activators and provide an influx of calcium into the cell [14].

Calcium-triggered apoptosis is a principle pathway that regulates cell growth and death [15]. Apoptosis is mainly but not exclusively triggered by caspases [16]. Breast cancer cells express various isoforms of caspases. Caspase-3 is present in MDA-MB-231, but not in MCF7 cells [17], which express other isoforms such as caspase-9 [18, 19].

To test the hypothesis that altered bioelectricity in cell membranes may be independent of breast cancer subtypes, we investigated the effects of voltage-gated potassium and calcium channel blockers on proliferation of two different breast cancer cell lines, MCF7 and MDA-MB-231. Particularly, we focused on cell death induced by verapamil, a clinically used calcium channel blocker in treatment of hypertension, and its underlying mechanisms.

## Methods

### Cell culture

Human breast adenocarcinoma cells (ER-positive MCF7 and triple-negative MDA-MB-231) were routinely cultured in Dulbecco’s modified eagle’s medium (DMEM, invitrogen) containing 10 % fetal bovine serum, 100 IU/mL penicillin, and 100 g/L streptomycin. Normal HMEC (ATCC) were grown in mammary epithelial cell basal medium (MEC), supplemented with MEC growth kit (ATCC). Cells were washed with PBS and media was replaced daily until 90 % confluence was reached. After addition of TEA or verapamil, cells were monitored every 24 h for cell growth and death.

### Whole-cell patch clamp measurement of resting membrane potential

For resting membrane potential measurement using whole-cell patch clamp technique, cells were transferred to polylysine-coated coverslips. These were placed in a lucite bath with the temperature maintained at 35–37 °C using a temperature control unit (TC2BIP, *Cell MicroControls*). Resting membrane potential (E_m_) was recorded using the whole cell patch clamp technique with an Axopatch–700B amplifier. E_m_ was measured with normal Tyrode’s and pipette solutions. The Tyrode’s solution contained (in mM): 143 NaCl, 5.4 KCl, 1.8 CaCl_2_, 0.5 MgCl_2_, 0.25 NaH_2_PO_4_, and 5 HEPES; pH was adjusted to 7.4 by NaOH. The pipette solution contained (in mM): 120 KCl, 1 CaCl_2_, 5 MgCl_2_, 5 Na_2_ATP, 11 EGTA, 10 HEPES, and 11 glucose; pH was adjusted to 7.3 by KOH.

### Live cell imaging

Cells in 6-well plates were imaged daily using a Zeiss fluorescent microscope equipped with an AxioCam HRc camera and AxioVision 4.6 imaging software.

### Quantification of cells using ImageJ

Fiji ImageJ was used for cell quantification. The image threshold range was set manually for all images using the ‘Dark Background’ setting. When using the ‘Analyze Particles’ plugin, a minimum size value of 0.001 pixels^2^ was set to prevent miscounting cell debris as live cells.

### Immunoblotting

Protein extracts were prepared after 90 % death was observed. Floating cells were pelleted from the media by centrifugation (3000×*g* for 5 min) and then resuspended in lysis buffer (fresh protease and phosphatase inhibitors (Sigma), 20 mM Tris, 150 mM NaCl, 10 mM EGTA and 10 mM EDTA at pH 7.4). Buffer was then added to culture dishes and a cell scraper was used to detach cells. The dishes were allowed to sit for 5 min before cellular debris was centrifuged out of solution. Supernatants were placed into new tubes and protein concentrations were recorded using Bradford’s method on an Eppendorf biophotometer.

For western blotting procedures, protein concentrations were normalized between samples to 20 μg and mixed with non-reducing lane marker (Thermo Fisher) with 5 % β-mercaptoethanol. After heating in a water bath to 95 °C for 5 min, samples were cooled to 4 °C then loaded into a 4–12 % bis–tris gels (invitrogen). Electrophoresis was carried out at 80 V for 30 min then 160 V for the remainder.

Proteins were transferred to pre-wetted nitrocellulose membranes (0.2 μm pore size) at 30 V for 1 h. Blots were blocked with 3 % bovine serum albumin (BSA) in tris-buffered saline with 0.1 % tween-20 (TBS-T) for 1 h before primary caspase-3 or caspase-9 antibody (1:1000 dilution; cell signaling) was added on a shaker at 4 °C overnight. Primary antibody solution was replaced with fresh 3 % BSA in TBS-T containing secondary antibodies at 1:10,000 dilution for 1 h at room temperature on a shaker. After five washes with TBS-T, blots were developed with a standard ECL kit (Life Technologies) on x-ray film or using a G:BOX digital imaging system (Syngene).

### Statistical analysis

Data were presented as mean ± SEM. Student’s *t* test was used to calculate the statistical significance between two groups. ANOVA was used to calculate the statistical significance among multiple groups. Data were considered as statistically significant when p < 0.05.

## Results

### Bioelectricity and cell growth of normal and tumor mammary epithelial cells

**Fig. 1:**
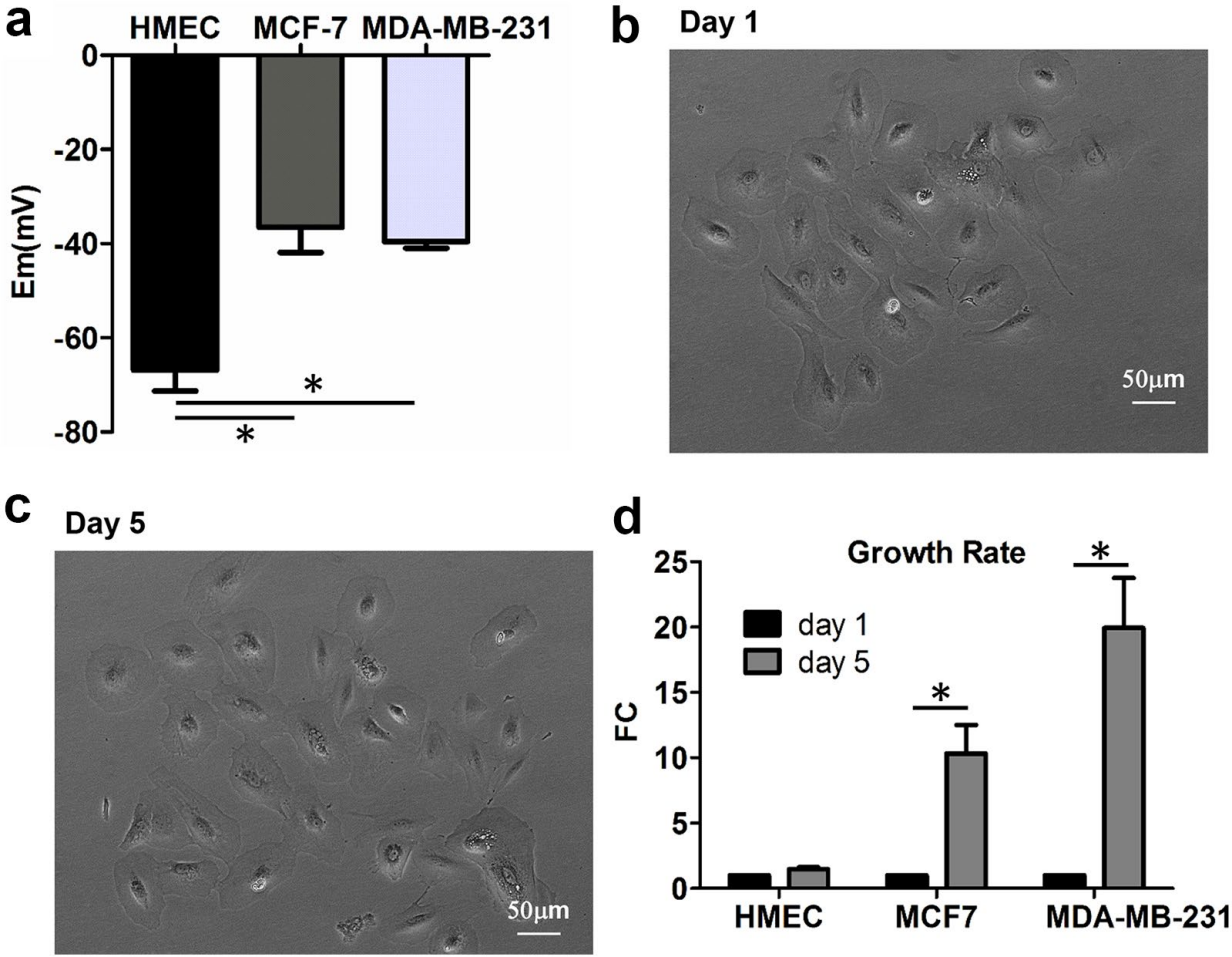
Bioelectricity and cell growth. **a** Resting membrane potential (E_m_) in HMEC, MCF7, and MDA-MB-231, n = 10 for each group. **b** HMEC at day 1. **c** HMEC at day 5. **d** Cell growth at day 5 compared to day 1 (normalized) for HMEC, MCF7, and MDA-MB-231. n = 4 for each group. *FC* fold change. *Asterisk* indicates statistical significance

Figure 1a compares the resting membrane potential (E_m_) in HMEC, MCF7, and MDA-MB-231 cells. MCF7 cells are 30.4 mV more depolarized in comparison to HMEC cells (E_m__MCF7 = −36.5 ± 5.4 mV, E_m__HMEC = −66.9 ± 4.4 mV, n = 8, p < 0.005). MDA-MB-231 cells are 27.3 mV more positive compared to HMEC (E_m__MDA-MB-231 = −39.5 ± 5.4 mV, E_m__HMEC = −66.9 ± 4.4 mV, n = 8, p < 0.001). HMEC at days 1 and 5 are shown in Fig. 1b and c, respectively. Compared to day 1, cells grew 1.47 ± 0.16-fold in HMEC (n = 4, p < 0.05), 10.33 ± 2.19-fold in MCF7 (n = 4, p < 0.05), and 19.93 ± 3.83-fold in MDA-MB-231 (n = 4, p < 0.05) (Fig. 1d).

### Stimulation of breast cancer cell growth by membrane depolarization

**Fig. 2:**
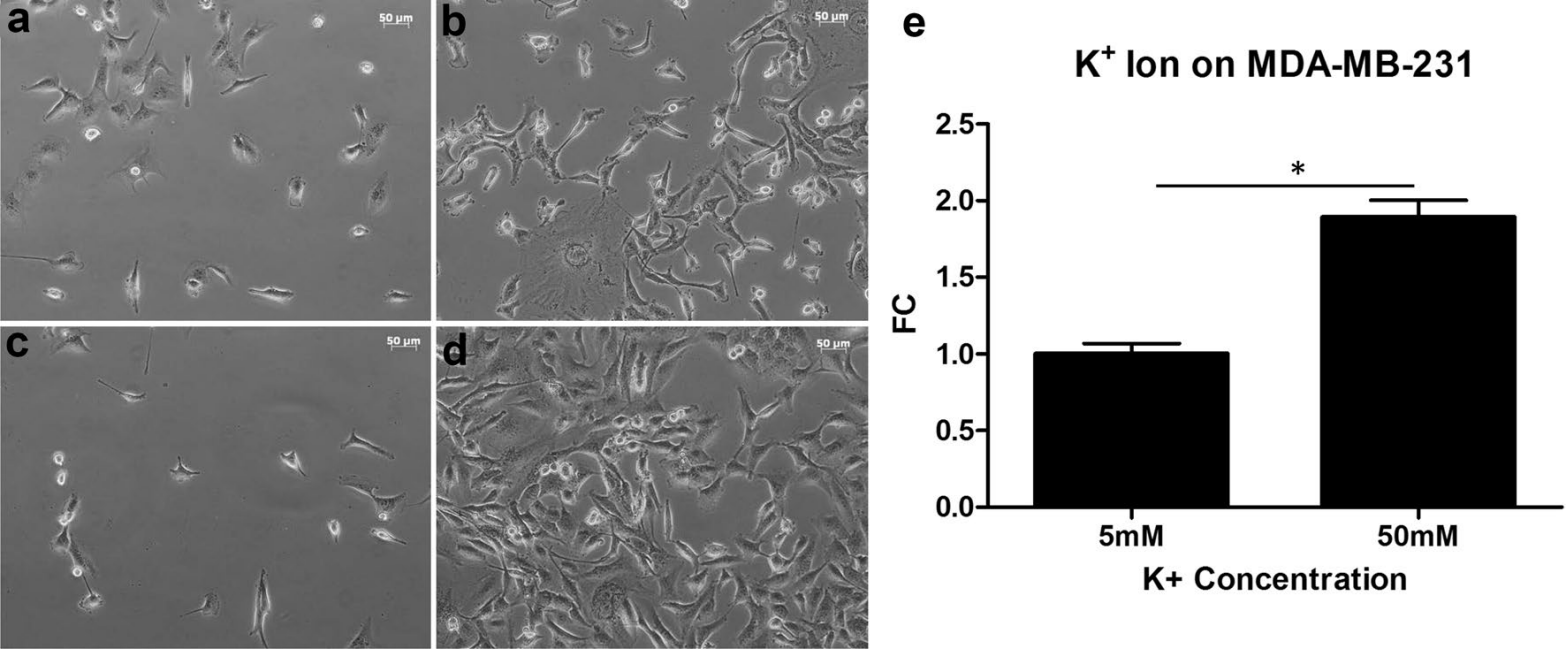
Effect of increased K^+^ concentration on MDA-MB-231 cell growth. MDA-MB-231 cell growth in the presence of 5 mM K^+^ at day 1 (**a**) and day 6 (**b**), and in the presence of 50 mM K^+^ at day 1 (**c**) and day 6 (**d**). Averaged cell growth of MDA-MB-231 cell populations in different concentrations of K^+^ (E). *FC* fold change. *Asterisk* indicates statistical significance

The normal potassium concentration in standard cell culture medium (DMEM) is 5 mM. Altering potassium concentration in DMEM changes membrane potential, which affected growth of MDA-MB-231 cells (Fig. 2). We started cell culture with approximately the same number of cells on day 1 under different potassium concentrations (Fig. 2a, 5 mM, Fig. 2c, 50 mM). After 5 days, cells grew significantly more in culture containing 50 mM K^+^ ions (Fig. 2d) than in culture containing 5 mM K^+^ (Fig. 2c). On average, the growth rate was increased by 1.89 ± 0.07-fold in 50 mM K^+^ medium than in 5 mM K^+^ medium (growth rate was normalized to 5 mM K^+^, n = 3, p < 0.01) after 5 days of culture (Fig. 2e).

**Fig. 3:**
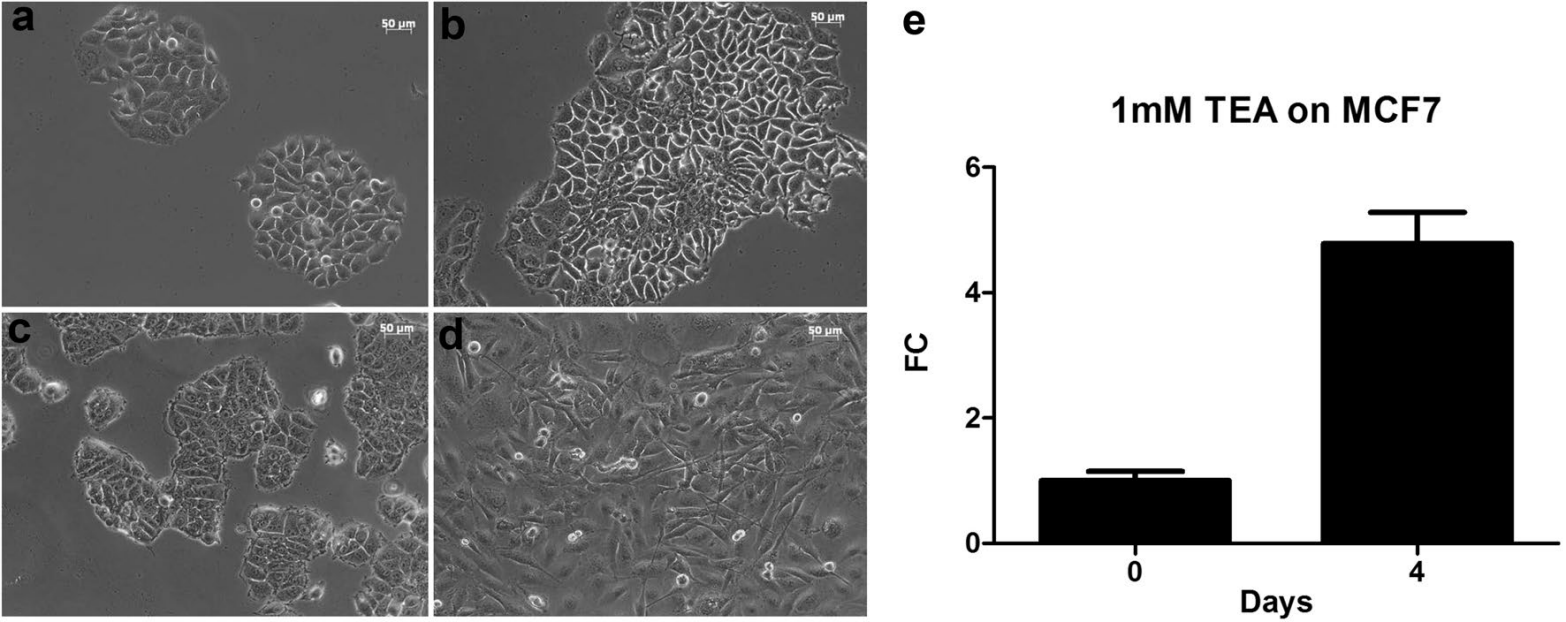
Effect of TEA on MCF7 cell growth. MCF7 cell growth in the presence of 1 mM TEA at day 0 (**a**) and day 4 (**b**), and in the presence of 1 mM TEA at day 0 (**c**) and day 4 (**d**) of growth. Averaged cell growth of MCF7 cells with 1 mM TEA at days 0 and 0 (**e**). *FC* fold change

Voltage-gated potassium channels control the plasma membrane potential and blocking these channels induces membrane depolarization [20]. We used a non-selective potassium channel blocker, TEA, to study the effect of depolarization on growth of MCF7 cells. In the absence of TEA, cells grew 225 % after 4 days in culture (Fig. 3a, b) (n = 128.2 ± 12 at day 0, n = 416.7 ± 22 at day 4, p < 0.01, n = 3). In the presence of 1 mM TEA, cells grew 4.78 ± 0.50 fold at day 4 compared to day 0 (normalized) (Fig. 3c–e, n = 3, p < 0.01). These results indicate that changes in membrane potential have significant effects on growth rate of breast cancer cells.

### Calcium as a key modulator in breast cancer cell growth

**Fig. 4:**
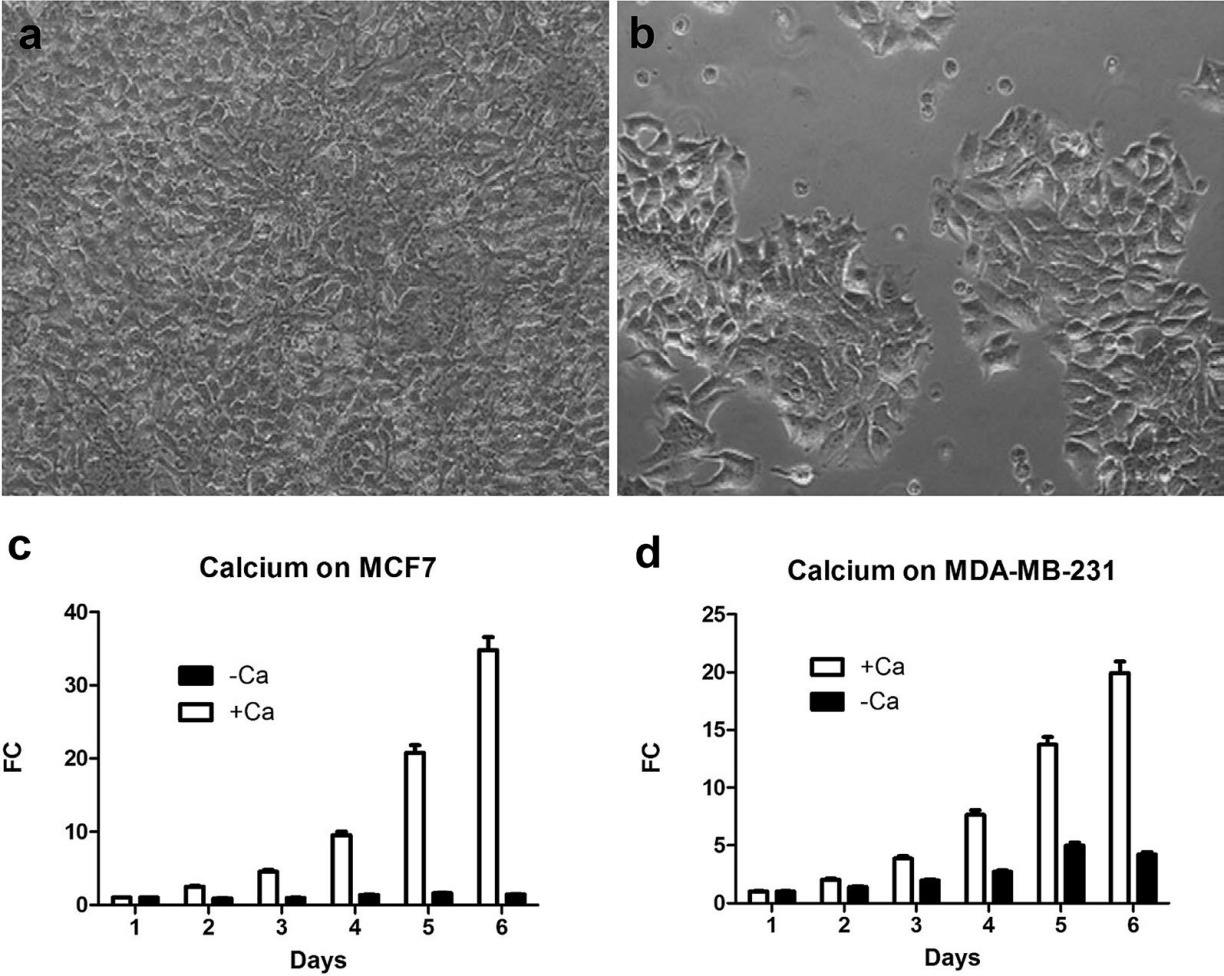
Effect of Ca^2+^ on MCF7 and MDA-MB-231 cell growth. MCF7 cell growth in the presence of Ca^2+^ (**a**) and in the absence of Ca^2+^ (**b**). Averaged cell growth of MCF7 (**c**) and MDA-MB-231 (**d**) in the absence and presence of Ca^2+^. *FC* fold change

To understand how depolarized membranes can stimulate growth of breast cancer cells, we studied the effect of calcium ions on cell proliferation. After 5 days, MCF7 cells barely grew in the absence of Ca^2+^ ions in the culture medium (Fig. 4b) compared with cells growing to nearly 100 % confluence in medium that contains 2 mM Ca^2+^ ions (Fig. 4a). The average cell population growth in the absence and presence of Ca^2+^ ions is shown in 4C for MCF7 and in 4D for MDA-MB-231, respectively. Cell numbers at day 1 were normalized (Fig. 4c, d). In normal DMEM containing Ca^2+^, MCF7 cells grew 2.5 ± 0.07-fold at day 2, 4.56 ± 0.13 at day 3, 9.55 ± 0.28-fold at day 4, 20.78 ± 0.60-fold at day 5, and 34.83 ± 1.01-fold at day 6; and MDA-MB-231 cells grew 2.03 ± 0.06-fold at day 2, 3.87 ± 0.11-fold at day 3, 7.66 ± 0.22-fold at day 4, 13.71 ± 0.40-fold at day 6, and 19.93 ± 0.58-fold at day 6. In the absence of external Ca^2+^, cells barely grew (Fig. 4c, dark bars) or grew in a significantly slower rate compared to that with Ca^2+^ (Fig. 4d, dark bars). These results indicate the essential requirement of external Ca^2+^ ions during growth of breast cancer cells.

**Fig. 5:**
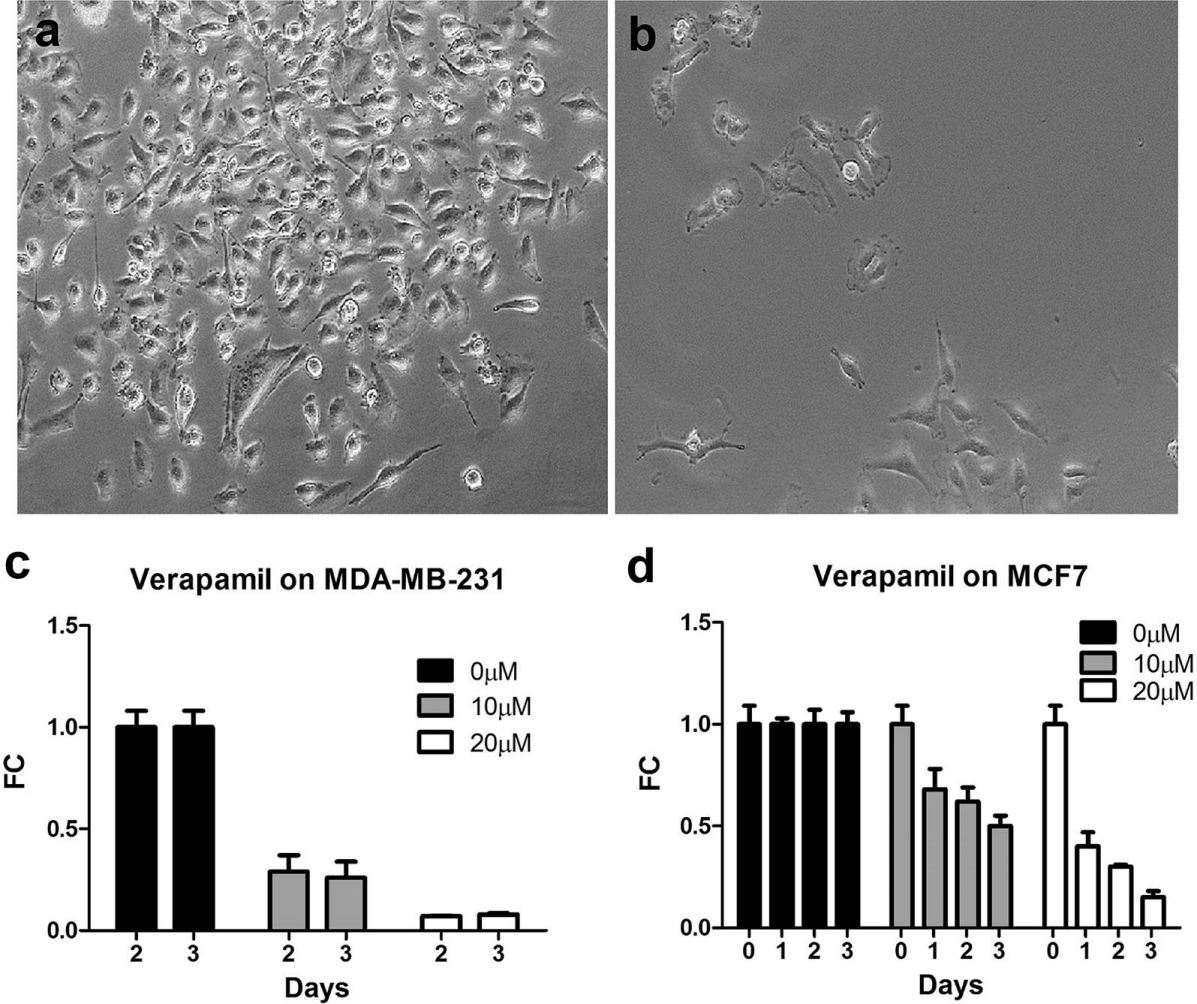
Effect of verapamil on MCF7 and MDA-MB-231 cell growth. MDA-MB-231 cell growth in the presence of 10 μM verapamil at day 5 (**a**) and day 6 (**b**). Verapamil was added on day 5. Averaged cell growth of MDA-MB-231 (**c**) and MCF7 (**d**) for different concentrations of verapamil. Growth rate was normalized at 0 μM verapamil. *FC* fold change

External Ca^2+^ ions can be blocked from entering the cell specifically through voltage-gated calcium channels [11] with verapamil. Figure 5a shows MDA-MB-231 cells grown in normal medium without verapamil. After 1 day of incubation with 10 μM verapamil, cell number dramatically decreased (Fig. 5b). Figure 5c shows the growth of MDA-MB-231 cells at day 2 and 3 under different concentrations of verapamil. Compared to the absence of verapamil (0 μM), verapamil inhibited cell growth by 74 % at 10 μM and 92 % at 20 μM, respectively (n = 3, p < 0.01). Figure 5d shows inhibited growth of MCF7 cells by verapamil. At day 3, verapamil inhibited cell growth by 49 % at 10 μM and 85 % at 20 μM, respectively (n = 3, p < 0.05).

### Mechanism of verapamil-induced death in breast cancer cells

One common cellular mechanism associated with calcium in cell death is the caspase signaling pathway [15]. Among the many caspases in breast cancer cells, we focused on caspase-3 not only because it is the principle caspase in caspase family [21], but also since it is present in MDA-MB-231 but not in MCF7 cells [17].

**Fig. 6:**
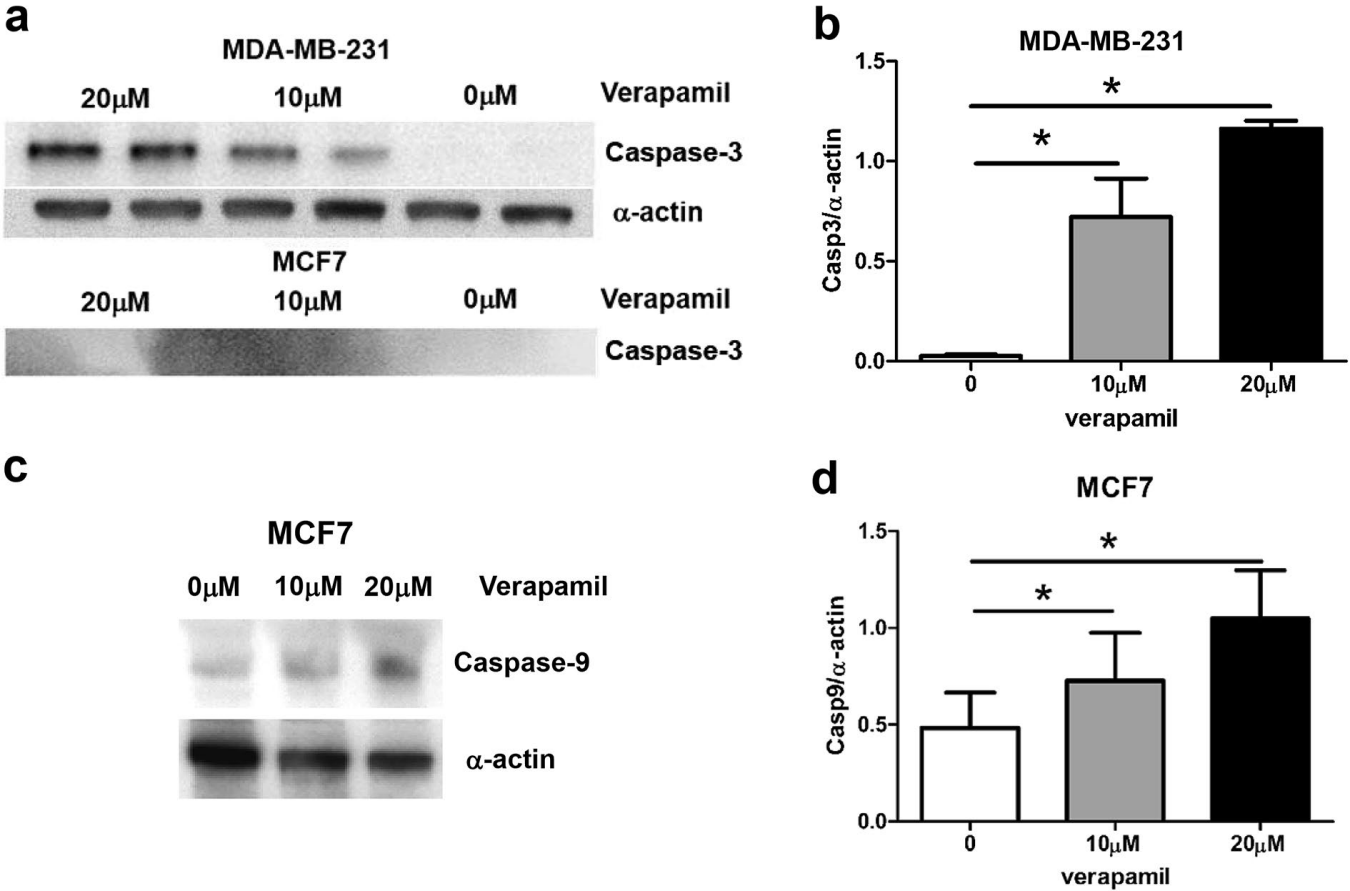
Verapamil-induced caspase expression in MCF7 and MDA-MB-231. Representative caspase immunoblots of MDA-MB-231 and MCF7 cells under different concentrations (0, 10, and 20 μM) of verapamil. **a** Caspase-3 expressed in MDA-MB-231 but not in MCF7 cells. **b** Verapamil concentration-dependent expression levels of caspase-3 in MDA-MB-231. **c** Caspase-9 expression in MCF7 cells. **d** Verapamil concentration-dependent expression levels of caspase-9 in MCF7. Alpha-actin was used as a loading control. *Asterisk* indicates statistical significance

Figure 6 shows the representative western blots for caspase levels in MCF7 and MDA-MB-231 cells. The levels of protein expression of caspase-3 were increased by verapamil in a concentration-dependent manner in MDA-MB-231, whereas no caspase-3 protein expression was detected in MCF7 cells (Fig. 6a). In comparison to the absence of verapamil, α-actin normalized caspase-3 levels are increased by 28-fold in 10 μM verapamil (0 μM = 0.026 ± 0.007, 10 μM = 0.72 ± 0.19, n = 3, p < 0.05) and 45-fold in 20 μM verapamil (0 μM = 0.026 ± 0.007, 20 μM = 1.16 ± 0.04, n = 3, p < 0.001) (Fig. 6b). In MCF7 cells the caspase-9 levels are increased after verapamil treatment (Fig. 6c). In comparison to the absence of verapamil, α-actin normalized caspase-9 levels are increased by 1.9-fold in 10 μM verapamil (0 μM = 0.42 ± 0.12, 10 μM = 0.79 ± 0.20, n = 3), but insignificantly (p > 0.05), and 2.6-fold in 20 μM verapamil (0 μM = 0.42 ± 0.12, 20 μM = 1.11 ± 0.19, n = 3, p < 0.05) (Fig. 6d).

## Discussion

Depolarized membrane potential has recently been recognized as a potential novel bioelectrical marker of cancer cells [22, 23]. In this study, we explored the notion that the intrinsic association of membrane potential and breast cancer growth is independent of breast cancer subtypes. We tested the idea using two different types of human breast cancer cell lines, an ER+ MCF7 and a triple-negative MDA-MB-231. We attempted to address a specific question: if membrane depolarization is indeed associated with cell growth of breast cancer cells, what mediates the membrane depolarization and proliferation of breast cancer cells? Alterations of ion channel expression and activities are associated with the initiation, proliferation, and metastasis of cancer cells [5, 8]. Voltage-gated calcium channels are present in human breast cancer cells but not in normal human mammary epithelial cells [13]. Therefore, we focused on voltage-gated potassium and calcium channels as potential underlying mechanisms that link membrane depolarization and breast cancer growth.

We showed that membrane potentials of MCF7 and MDA-MB-231 are depolarized in comparison to that of normal human mammary epithelial cells. HMEC grew much slower compared to MCF7 and MDA-MB-231. These results are consistent with previous reports that demonstrated the association between membrane potential and breast cancer patients [10].

Second, we showed that membrane depolarization, induced by increasing external potassium concentration or blocking voltage-gated potassium channels, stimulated growth of MCF7 and MDA-MB-231 cells. Third, we demonstrated that calcium is essential for growth of MCF7 and MDA-MB-231 since removing external calcium led to death of both types of cells. These results are consistent with the essential role of calcium in cell growth and cancer development [24]. Fourth, we observed reduced proliferation and death of MCF7 and MDA-MB-231 cells by verapamil, reinforcing the role of calcium entry through voltage-gated calcium channels during the growth of breast cancer cells. We noticed that verapamil inhibited growth of MDA-MB-231 to the similar degree at days 2 and 3 (Fig. 4c), but it appears there is time-dependent inhibition of verapamil on cell growth of MCF7 (Fig. 4d).

To understand the underlying mechanisms of verapamil-induced cell death in breast cancer cells, we explored the potential role of caspase signaling, known to be integral in calcium- induced apoptosis [15]. We found that the caspase-3 levels in MDA-MB-231 cells were significantly increased during chronic exposure to verapamil. In MCF7, no protein expression of caspase-3 was detected (Fig. 6a). Rather, caspase-9 protein levels are increased in response to verapamil treatment (Fig. 6c). It is imperative to emphasize that caspase-9 may not be the main mechanism that mediates the apoptotic effect of verapamil on MCF7, since the increase in its protein levels is small (Fig. 6d) compared to verapamil-induced increase in caspase-3 in MDA-MB-231 (Fig. 6b). Other caspases, such as caspase-6 and caspase-7, might be more important in mediating the verapamil-induced cell death in MCF7 [18, 19]. Additionally, there exists caspase-independent apoptosis in both MCF7 and MDA-MB-231 cells [25–27]. Therefore, the exact mechanisms that mediate verapamil-induced apoptosis require more investigation.

A recent study demonstrated that depolarized membrane potential increases the clustering of phosphatidylserine lipids and K-Ras, promoting RAF-MAPK signaling, which is known to induce proliferation of cancer cells [22, 28, 29]. This mechanism has yet to be validated with regard to intracellular calcium homeostasis in the breast cancer cell culture models used in our study.

## Conclusions

Collectively, we showed that membrane potential depolarization occurs in breast cancer cells, independent of subtypes. Verapamil, a voltage-gated calcium channel blocker which is clinically used to treat hypertensive patients can induce cell death in both MCF7 and MDA-MB-231. However, cellular mechanisms that mediate verapamil-induced inhibition of breast cancer cells are different and likely subtype-specific.

## Abbreviations

TEA: tetraethylammonium
ER: estrogen receptor
PR: progesterone receptor
HER2: human epidermal growth factor receptor 2
IDC: invasive ductal carcinoma
HMEC: human mammary epithelial cells
DMEM: Dulbecco’s modified eagle’s medium
RAF: rapidly accelerated fibrosarcoma
MAPK: mitogen-activated protein kinase
BSA: bovine serum albumin
TBS-T: tris-buffered saline with 0.1 % tween-20
